# Prognostic value of HPV-PCR, p16 and p53 immunohistochemical status on local recurrence rate and survival in patients with vulvar squamous cell carcinoma

**DOI:** 10.1007/s00428-023-03690-8

**Published:** 2023-11-08

**Authors:** A. W. Pouwer, N. C. te Grootenhuis, F. Hinten, G. H. de Bock, A. G. J. van der Zee, W. J. G. Melchers, M. H. M. Oonk, J. A. de Hullu, H. Hollema, J. Bulten

**Affiliations:** 1 Department of Obstetrics and Gynaecology, Radboud University Medical Center, Nijmegen, The Netherlands; 2 Department of Obstetrics and Gynaecology, University Medical Center Groningen, University of Groningen, Groningen, The Netherlands; 3 Department of Epidemiology, University Medical Center Groningen, University of Groningen, Groningen, The Netherlands; 4 Department of Medical Microbiology, Radboud University Medical Center, Nijmegen, The Netherlands; 5 Department of Pathology, University Medical Center Groningen, University of Groningen, Groningen, The Netherlands; 6 Department of Pathology, Radboud University Medical Center, Nijmegen, The Netherlands

**Keywords:** Vulvar carcinoma, HPV, p16, p53, Local recurrence, Survival

## Abstract

The primary aim of this study was to assess the association between human papilloma virus (HPV) and p53 expression and local recurrence (LR), disease specific survival (DSS), and overall survival (OS) in patients with vulvar squamous cell carcinoma (VSCC). Secondary, the accuracy of p16 immunohistochemistry for HPV status was assessed. The tumor tissue of 255 patients, surgically treated for primary unifocal VSCC between 2000 and 2010, was analyzed. HPV-PCR and P16 and p53 immunohistochemical stainings were performed. All histologic slides were independently reviewed by two expert gyneco-pathologists. Time to first LR, DSS, and OS for the variables p16, p53, and HPV-PCR were compared using univariable and multivariable Cox-regression analyses. In 211/255 (83.5%) patients, HPV-PCR was negative. The local recurrence rate was significantly lower in patients positive with HPV-PCR (10-year LR rate 24.6%) versus negative tumors (47.5%), *p* = 0.004. After multivariable analyses, this difference remained significant (HR 0.23 (95% CI 0.08–0.62) *p* = 0.004). There was no difference in LR rate correlated to the p53 expression. DSS and OS did not significantly differ after multivariable analyses for all different subgroups. Sensitivity and specificity of p16 staining for presence of HPV detected by HPV-PCR were 86.4% and 93.8%, respectively. In conclusion, patients with HPV-negative VSCCs have significantly more LR compared to patients with HPV-positive VSCCs, and p16 immunohistochemistry is a reliable surrogate marker for HPV status. No relevant subgroup for LR or survival based on HPV/p53 status could be identified. We advise to perform an HPV-PCR or p16 IHC staining in all patients with VSCC.

**Supplementary Information:**

The online version contains supplementary material available at 10.1007/s00428-023-03690-8.

## Introduction

Vulvar squamous cell carcinoma (VSCC) accounts for over 80% of the vulvar cancers [1]. There are currently two different etiologic types of VSCC recognized, each with their own precursor lesions. The first, most common, type often leads to differentiated keratinizing VSCC related to lichen sclerosus or other chronic inflammatory dermatosis. This type commonly has differentiated vulvar intraepithelial neoplasia (dVIN) as a precursor lesion and occurs mainly in elderly women. The second type is related to high-risk human papillomavirus (HPV) infections. The described prevalence of HPV-related VSCC ranges between 3.3 and 76.5%, with a pooled prevalence of 33.7% as described by Zhang et al*.* [2]. It consists of mainly non-keratinizing carcinomas, with high-grade intraepithelial squamous lesions (HSIL) identified as its precursor [3–5].

Currently, morphologic criteria of the carcinoma and its precursor lesion on hematoxylin and eosin (H&E) stained slides are used in daily practice to classify non-HPV- and HPV-related VSCC, without routine testing for HPV presence. However, morphologic characteristics do not allow definite differentiation between HPV and non-HPV-related VSCC [6–8]. HPV-PCR is a more expensive and labor-intensive technique to determine the HPV-status of VSCC compared to p16 IHC. Immunohistochemistry (IHC) of p16 is accepted as a surrogate marker for the presence of HPV. However, only limited data from small studies in vulvar cancer are available, and these show unequivocal results [8–10].

The most common mutated gene in HPV-negative VSCC is TP53, but the prognostic value of TP53 mutations is unclear, especially in relation to local recurrence of VSCC. Scheistrøen et al. [11] reported p53 mutation in 55% of the included VSCC, which was associated with a reduced 5-year survival compared to p53-negative VSCC, and disease-related survival was not influenced. Hay et al. [12] showed that p53 mutation was associated with an increased recurrence rate and disease-specific mortality. A review of the literature [13] concluded that p53 mutation is associated with reduced 5-year overall survival; however, only small cohort studies were included, and no data regarding local recurrence was available. A single study reported the relation between p53 and local recurrence; they found a significant lower recurrence free survival within 2 years after treatment in patients with HPV-related VSCC compared to non-HPV-related/p53 wildtype or non-HPV-related/p53-mutated VSCC [14].

Nooij et al. suggested, based on the assessment of molecular alterations, a third etiologic pathway of VSCC, linked to the HPV status and p53 status [14]. The three suggested subtypes are (1) HPV-related, (2) non-HPV-related and p53 wildtype, and (3) non-HPV-related p53 mutated. TP53 mutation status can be assessed by next-generation sequencing and/or p53 IHC. Pattern-based p53 IHC reflects TP53 mutations in VSCC with a sensitivity of 95.3% and specificity of 100% [15]. Recently six major p53 IHC patterns were identified and reported to be consistent with a TP53 mutation in 95% of the VSCC [16]. In patients treated for early-stage VSCC, local recurrences are reported in up to 40% of this groups within 10 years after primary treatment, with significantly reduced disease specific survival [17, 18]. Data regarding the prognostic value, in terms of local recurrence and survival, of p53 is limited. It has clinical importance to select high-risk patients for local recurrence and/or survival to be able to optimize diagnosis, treatment, and follow-up in the future.

The primary aim of this study was to determine the association between HPV- and p53-status, local recurrence, disease specific survival (DSS), and overall survival (OS). Secondly, we determined the accuracy of p16 IHC for the prediction of HPV-status.

## Methods

This study is reported in accordance with the Reporting Recommendations for Tumor Marker Prognostic Studies (REMARK) [19].

### Patients

Paraffin-embedded tumor specimens and clinical and follow-up data were collected from patients with primary squamous cell carcinoma of the vulva from two expert center: the University Medical Center Groningen (UMCG) and Radboud University Medical Center (Radboudumc). Consecutive patients treated from January 2000 until December 2010 were selected for this study. Inclusion criteria were primarily surgically treated for squamous cell carcinoma of the vulva, and 18 years or older at time of diagnosis and available tissue blocks. Exclusion criteria for this study were multifocal disease, neo-adjuvant radio- and/or chemotherapy, and palliative treatment. This study cohort was previously published [20].

### Data handling

Data from patients treated for VSCC in both treatment centers are routinely stored in a database. We searched the Dutch nationwide registry of histopathology and cytology (PALGA) for additional patients to enrich the database. PALGA is the nationwide network and registry of histo- and cytopathology in The Netherlands and forms the basis for the national cancer registry [21]. Follow-up and recurrence data were collected from patient charts and were retrospectively added to the database.

The patient data were stored in an electronic database (Castor EDC) using study-specific numbers to guard the patient identities [22]. Only two data managers, for each treatment center separately, had access to the code list and were able to relate the specific codes to the identifying patient data. Because of these procedures and the fact that patients did not object against the use of their clinical data or tumor material, no further patient or Institutional Review Board approval was needed, according to the Dutch law applicable during this data collection.

### Specimen characteristics

#### Histopathologic review

All formalin-fixed and paraffin-embedded (FFPE) and hematoxylin and eosin (H&E) stained slides were reviewed by two independent expert gyneco-pathologists (JB and HH) for the presence of VSCC and its histopathological characteristics. The following histopathologic characteristics were assessed: tumor-free margin distance (after re-excision if performed), tumor diameter, depth of invasion, the presence of LVSI, growth pattern (spray, invasive, confluent, or mixed) [23], and grade of differentiation (good, moderate, or poor) [24]. The pathologists were blinded for the results of p16, p53, and/or HPV status.

### Assay methods

#### P16 and p53 immunohistochemistry

IHC was used to detect p16 and p53 expression of the tumor. Tissue sections of the archival FFPE samples were stained. IHC for p16 was performed using the automated Ventana XT system; the reaction was developed using CINtec® p16 histology kit or p16 E6H4 ready to use antibody from Roche, as described previously [3, 4]. For p53 IHC, p53 DO-1 ready to use antibody from Roche (BP 53_11 Ventana ready to use) was used in the automated Ventana XT system. Scoring of the p16 and p53 expression was independently performed by two expert gyneco-pathologists. All discrepant scores were reviewed during a consensus meeting.

Both the p16 nuclear and cytoplasmatic p16 staining were considered positive. The results were reported in a semi-quantitative fashion: negative if less than 25% of the cells had nuclear or cytoplasmic staining, and positive if more than 75% of the cells showed nuclear or cytoplasmic block staining [3]. Cases in which 25–75% of the cells were stained diffuse positive were considered borderline cases and discussed in the consensus meeting, where in all cases, consensus was reached if the cases should be marked as positive or negative.

P53 IHC scoring was reported in three different categories and in line with the current literature [16]. Samples were scored p53 wildtype (p53wt) if staining of tumor nuclei was normal and comparable to the adjacent normal epithelium. Diversity in staining pattern in basaloid cells was also scored as wildtype; moderate to strong nuclear p53 staining in the parabasal layers, with notable basal sparing, is recognized as mid-epithelial pattern. Three patterns were defined as mutated p53 staining (p53mt):

1. First, if more than 80% of the tumor nuclei were strongly stained and also included stronger staining in the basal layer cells (some HPV-positive cases may show a high expression of p53 but the basal layer in HPV-positive cases is always negative) or more than 80% of the tumor nuclei were strongly stained and had strong parabasal extension. Areas with severe cornification were not included in the score because only vital tumor cells with an intact nucleus were taken into account.
2. Second, if no expression was seen in tumor nuclei but with a positive internal control in the normal epithelium showing a wild-type pattern, then the expression was scored as a 0 mutation.
3. Third, cytoplasmatic staining with or without nuclear staining with a positive internal control in the normal epithelium; however, this type of staining was not observed in our cohort.

#### HPV presence and genotyping

FFPE tissue was treated with proteinase K solution and incubated overnight. DNA was isolated from all tissues using MagNA Pure (Roche, Bazel, Switzerland). The purified DNA was eluted in 50 µl TE-buffer. For detection and genotyping of HPV, broad spectrum HPV amplification was performed using the short-PCR-fragment line probe assay (SPF10-LiPA25; Labo Bio-medical Products B.V. Rijswijk, The Netherlands). This assay amplifies a small fragment of 65 bp from the L1 open reading frame and allows detection of a broad range of mucosal HPV genotypes (HPV 6, 11, 16, 18, 31, 33, 34, 35, 39, 40, 42, 43, 44, 45, 51, 52, 53, 54, 56, 58, 59, 66, 68 or 73, 70, and 74) with high analytical sensitivity. B-globin qPCR was used to evaluate DNA quality and PCR inhibition. Each isolation and PCR run contained HPV-positive and HPV-negative controls. The combined HPV SPF10 PCRDEIA- LiPA25 system for detection and genotyping of HPV has been described in detail elsewhere [25–27]. Only high-risk HPV-positive VSCC samples were considered HPV-positive.

### Study design

#### Endpoints

The primary endpoints were time to local recurrence, disease specific survival (DSS), and overall survival (OS) in relation to the determinants p16, p53, and HPV status. Sensitivity, specificity, positive predictive value (PPV), and negative predictive value (NPV) were calculated for p16 IHC to determine HPV status (HPV-PCR golden standard).

#### Definitions

Local recurrence was defined as any newly diagnosed invasive squamous cell carcinoma on the vulva, and time to local recurrence was defined as the period of time in months from the date of primary surgery to the date at which recurrence was identified by histopathology. DSS was defined as survival from the date of primary surgery to the date of death due to VSCC. OS was defined as survival from the date of diagnosis to the date of death (any cause) or the date of last follow-up. If there was no event (local recurrence, DS-death, or death), the follow-up ended at the date of the last hospital visit. Patients were reported lost to follow-up if no information on the last 24 months was available at the time of data collection. Follow-up data were collected until January 1, 2018.

A positive p16 staining was considered such if HPV was detected by HPV-PCR. Negative p16 staining was considered such if no HPV was detected by HPV-PCR.

### Statistical analysis

The sensitivity, specificity, PPV, and NPV of p16 for the presence of HPV determined by HPV-PCR were calculated, and the Clopper-Pearson exact method was used to determine the 95% confidence intervals. Associations between clinico-/pathological characteristics of the tumor or patients and HPV, p16, p53, or HPV/p53 status were performed with chi-square or Fisher’s exact test. Mann–Whitney *U* test (for non-normal distributed variables) and Kruskall-Wallis test were (normal distributed variables) used for the comparison between continuous variables and HPV, p16, p53, or HPV/p53 status. Local recurrence, DSS, and OS rates were estimated using the Kaplan–Meier method, and univariable cox-regression analyses were performed to evaluate the effect of p16, p53, and HPV status (negative/positive) on time to local recurrence, DSS, and OS. In this way, hazard ratios (HRs) and the related 95% confidences intervals (95% CIs) were determined. Multivariable Cox regression analyses were performed to correct for associations between clinico-/pathological characteristics of the tumor or patients and the tested subgroup groups in the survival analyses. Variables that had a *p*-value < 0.05 in the univariable analyses were incorporated in a multivariable Cox-regression analysis. The following subgroups were defined based on HPV and p53 status: (1) HPV-p53Wt, (2) HPV-p53Mut, (3) HPV + p53Wt, (4) HPV + p53Mut. Differences were considered statistically significant at *p* < 0.05. Data analysis was performed using SPSS version 23.0 (SPSS Inc. Armonk, USA).

## Results

In total, 287 patients fulfilled the inclusion criteria of our study. Of 32 patients (11%), no tumor tissue was available for p16 and p53 staining and HPV PCR. Therefore, we analyzed the data of 255 patients. The median age of all included patients was 73 years (range 26–100); FIGO stage IA-IVA were represented. See Table 1 for patient and tumor characteristics. Median follow-up was 83 months (range 0–202), and 19 (7.5%) patients were lost to follow-up within 24 months of primary treatment.

**Table 1:** Patient and tumor characteristics (*N* = 255)

	Median (range)	*N* (%)
Age	73 (26–100)	
FIGO 2009 stage		
IA		7 (2.7)
IB		115 (45.1)
II		5 (2.0)
IIIA		58 (22.7)
IIIB		13 (5.1)
IIIC		50 (19.6)
IVA		5 (2.0)
IVB		0
Unknown		2 (0.8)
Localization of carcinoma		
Labium		161 (63.1)
Perineum		19 (7.5)
Clitoris		52 (20.4)
Labium and clitoris		16 (6.3)
Labium and perineum		7 (2.7)
Event local recurrence		
Yes		83 (32.5)
No		172 (67.5)
Status		
Alive		141 (55.3)
Died of vulvar carcinoma		44 (17.3)
Died of intercurrent disease		60 (23.5)
Died of unknown cause		10 (3.9)
Tumor free margin distance (mm)	9.0 (0.0–130.0)	
Tumor diameter (mm)	30.0 (2.0–130.0)	
Depth of invasion (mm)	5.6 (0.6–25)	
LVSI		
No		202 (79.2)
Yes		49 (19.2)
Not assessed*		4 (1.6)
Growth pattern		
Spray		99 (38.8)
Invasive		87 (34.1)
Confluent		55 (21.6)
Mixed		6 (2.4)
Not assessed*		8 (3.1)
Grade of differentiation		
Good		75 (29.4)
Moderate		112 (43.9)
Poor		67 (26.2)
Not assessed*		1 (0.4)

One patient underwent a skinning vulvectomy because of vulvar intraepithelial neoplasia; coincidentally, this patient also had invasive squamous cell carcinoma that was excised sufficiently

*LVSI* lymph-vascular space invasion

^*^Not able to assess due to small tumors

### Association between p16 and HPV-PCR

As shown in Table 2, HPV was detected in 17.3% of the 255 tumors. Eighty percent of the 255 tumors were scored p16-negative and 20.0% p16-positive. In 74.5% of the p16-positive tumors, HPV was detected, and in 97.1% of the p16-negative tumors, no HPV was detected. The sensitivity of p16 IHC for the presence of HPV detected by p16 IHC was 86.4% (95% CI 72.7–94.8), and the specificity was 93.8% (95% CI 89.7–96.7), PPV 74.5% (95% CI 60.0–85.7), and NPV 97.1% (95% CI 93.7–98.9).

**Table 2:** Immunohistochemistry and HPV-PCR tumor characteristics

	*N* (%)
HPV status	
Negative	211 (82.7)
Positive	44 (17.3)
P16 status	
Negative	204 (80.0)
Positive	51 (20.0)
HPV/p16 status	
p16-negative	204
HPV-negative	198 (97.1)
HPV-positive	6 (2.9)
p16-positive	51
HPV-positive	38 (74.5)
HPV-negative	13 (25.5)
HPV subtype*	
16	32 (68.1)
18	6 (12.8)
31	1 (2.1)
33	2 (4.3)
39	1 (2.1)
51	1 (2.1)
52	2 (4.3)
53	1 (2.1)
67	1 (2.1)
p53 status	
0 mutation	9 (3.5)
Wild type	93 (36.5)
Mutation	126 (49.4)
No consensus	5 (2.0)
No tumor available	22 (8.6)
HPV/p53 status	
HPV-/p53Wt	66 (25.9)
HPV-/p53Mut	125 (49.0)
HPV + /p53Wt	27 (10.6)
HPV + /p53Mut	10 (3.9)
Missing	27 (10.6)

^*^One patient was positive for subtypes 16, 31, and 52; another patient was positive for subtypes 16 and 51

**Fig. 1:**
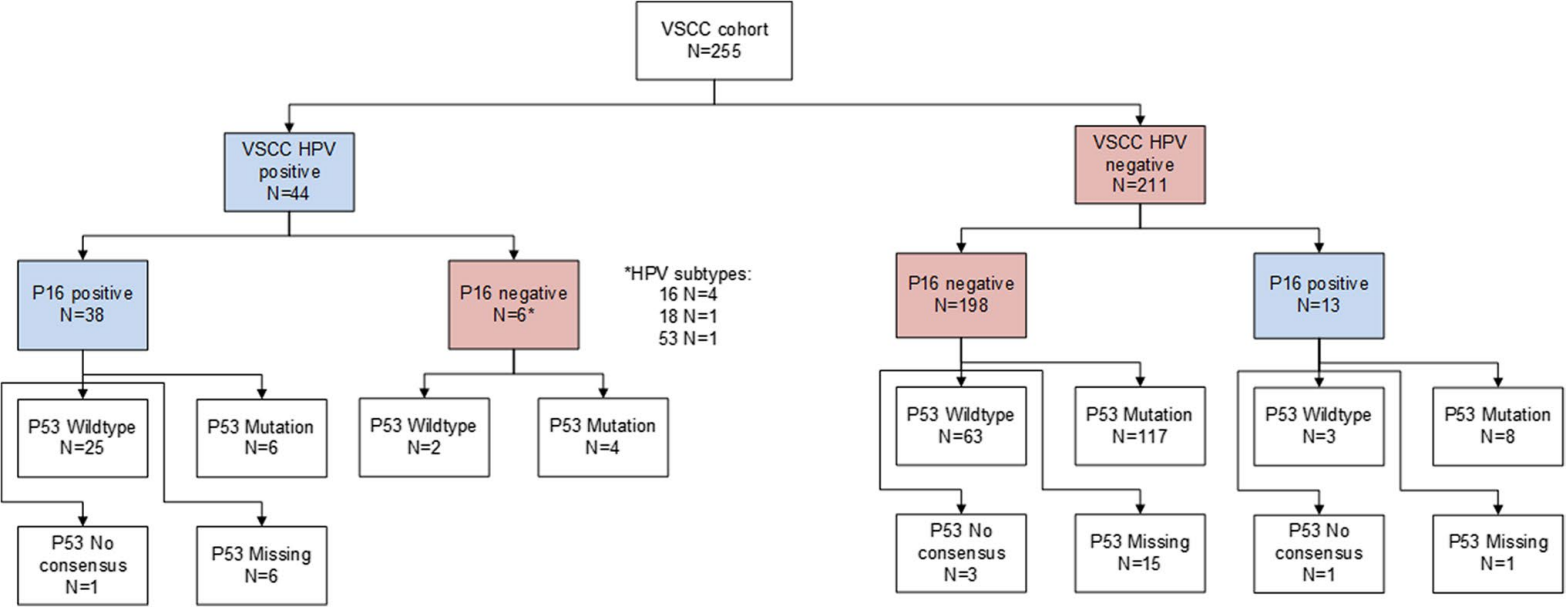
Flowchart regarding HPV, p16 and p53 status of the tumors; *VSCC* vulvar squamous cell carcinoma

In 36.5% of the tumors, p53 was scored as wildtype, whereas in 49.4% as p53 mutated. In 5 cases, no consensus could be reached; in 4 cases, p53 0 Mut versus Wt, and in one case, p53 Wt versus Mut. In 8.6% of the tumors, data were missing because no tumor tissue was available to score; see Table 2. Figure 1 displays the relations between HPV, p16, and p53 status of the tumors.

### Clinicopathological factors, local recurrence, and survival

Comparing HPV-positive versus -negative tumors, HPV-positive tumors were less invasive (median depth of invasion 4.1 versus 6.0 mm, *p* < 0.001) and were more confluent or invasive like in growth pattern (HPV-positive versus HPV-negative tumors, respectively (spray 23.3% versus 43.6%, invasive 41.9% versus 33.8%, and confluent 34.9% versus 19.6% *p* = 0.025). HPV-negative tumors were more often p53 mutated than HPV-positive tumors (125/191 (65.4%) versus 10/37 (25%) *p* < 0.001). We could not identify any differences between HPV-positive and -negative tumors in terms of tumor diameter, smaller margin distance, lymph vascular space invasion, or grade of differentiation. Patients with HPV-positive tumors were younger than patients with HPV-negative tumors; more specifically, mean age was 67 versus 74 years (*p* = 0.001). Also, patients with HPV-positive tumors had more early/stage tumors (FIGO stage IA/IB versus II, III, and IV) (*p* = 0.012).

**Fig. 2:**
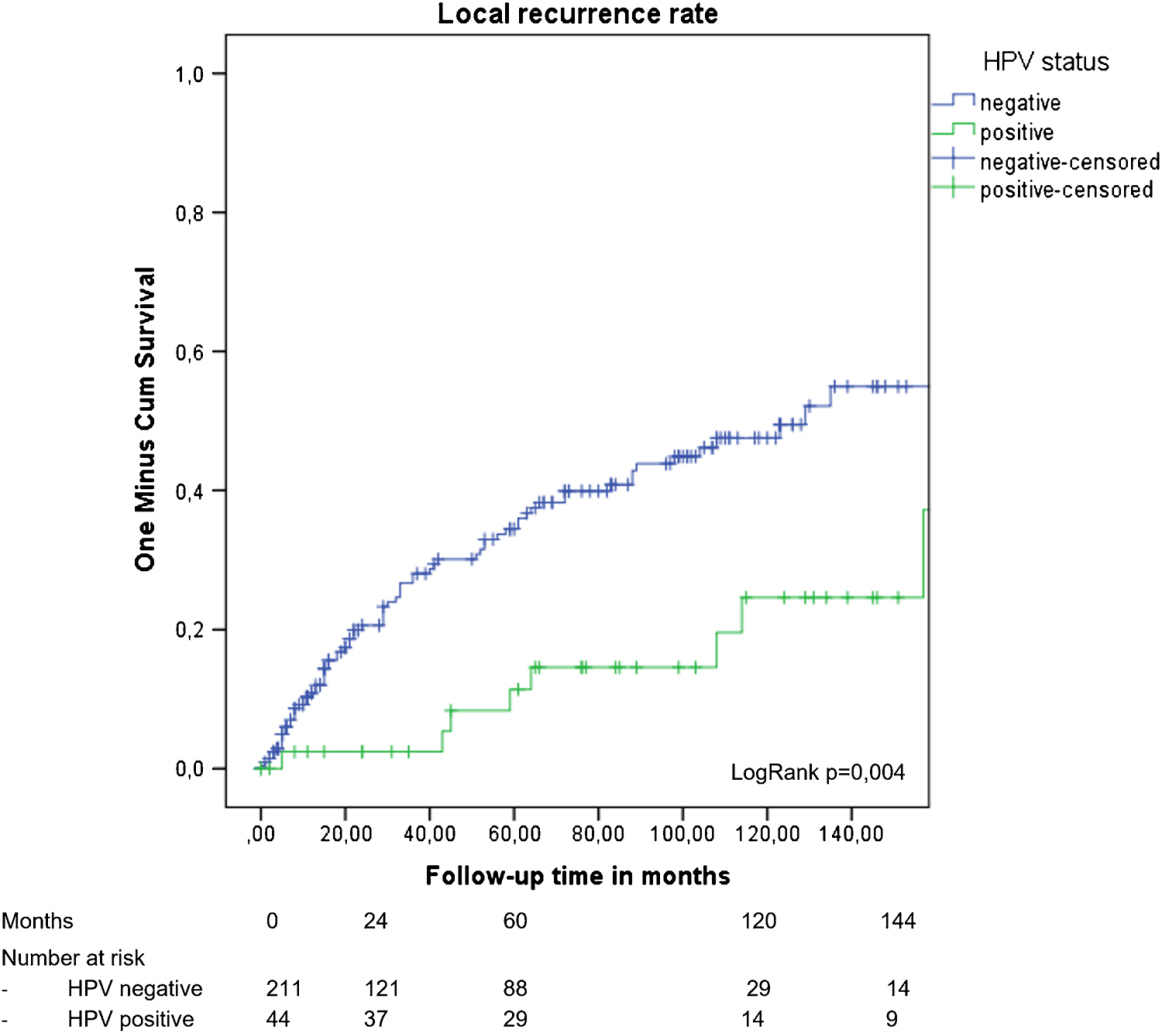
Local recurrence by HPV status; LogRank *p* = 0.004. HR 0.36 (95% CI 0.17–0.74 *p* = 0.006). Adjusted for age, FIGO stage, depth of invasion, growth pattern, and p53 status HR 0.23 (95% CI 0.08–0.62 *p* = 0.004)

The 5- and 10-year local recurrence rates were higher in patients with HPV-negative tumors compared to patients with HPV-positive tumors, 34.5% and 47.5% versus 11.4% and 24.6%, respectively, adjusted HR 0.23 (95% CI 0.08–0.62 *p* = 0.004) (adjusted for age, FIGO stage, depth of invasion and growth pattern, and p53 status) (see Fig. 2 and Table 3). No significant differences were found in DSS between patients with HPV-negative and -positive VSCC (adjusted HR 0.43 (95% CI 0.15–1.20) *p* = 0.108). For p16 IHC status, a higher survival was found for p16-positive tumor compared to p16-negative tumors in univariable analyses (HR 0.35 (95% CI 0.12–0.97) *p* = 0.043), which did not remain significant in multivariable analyses (see Table 3). Five- and 10-year OS were higher in patients with HPV-related VSCC compared to patients with non-HPV-related VSCC (80.9% and 71.0% versus 67.4% and 49.6%, respectively) in univariable analyses (HR 0.53 (95% CI 0.30–0.94) *p* = 0.031). Adjusted for age, FIGO stage, depth of invasion, growth pattern, and p53 status, this did not remain significant, HR 1.30 95% CI (0.66–2.54) *p* = 0.452).

**Table 3:** Survival data for p16, p53 and HPV status

	Local recurrence rate			Disease specific survival			Overall survival		
	Univariable KM (10-year rate %)	Univariable HR (95% CI)	Multivariable HR (95% CI)	Univariable KM (10-year rate %)	Univariable HR (95% CI)	Multivariable HR (95% CI)	Univariable KM (10-year rate %)	Univariable HR (95% CI)	Multivariable HR (95% CI)
p16									
Negative	48.7	1.0	1.0	78.0	1.0	1.0	49.6	1.0	1.0
Positive	24.2	0.39 (0.20–0.76)	0.36* (0.15–0.83)	91.7	0.35 (0.12–0.97)	0.61* (0.19–1.91)	67.2	0.59 (0.35–0.99)	1.22* (0.68–2.20)
	***p* = 0.004**	***p* = 0.005**	***p* = 0.017**	***p* = 0.034**	***p* = 0.043**	*p* = 0.391	***p* = 0.042**	***p* = 0.046**	*p* = 0.510
HPV-PCR									
Negative	47.5	1.0	1.0	79.0	1.0	1.0	49.6	1.0	1.0
Positive	24.6	0.36 (0.17–0.74)	0.23* (0.08–0.62)	89.7	0.43 (0.15–1.20)	0.72* (0.22–241)	71.0	0.53 (0.30–0.94)	1.30* (0.66–2.54)
	***p* = 0.004**	***p* = 0.006**	***p* = 0.004**	*p* = 0.097	*p* = 0.108	*p* = 0.596	***p* = 0.028**	***p* = 0.031**	*p* = 0.452
p53									
Wild type	36.8	1.0	1.0	85.2	1.0	1.0	64.6	1.0	1.0
Mutation	49.7	1.38 (0.86–2.21)	1.06# (0.64–1.77)	77.3	1.66 (0.84–3.28)	1.07# (0.51–2.22)	45.3	1.85 (1.21–2.84)	1.47# (0.92–2.34)
	*p* = 0.186	*p* = 0.186	*p* = 0.822	*p* = 0.144	*p* = 0.149	*p* = 0.866	***p* = 0.004**	***p* = 0.005**	*p* = 0.108
HPV/p53									
HPV-/p53Wt	46.8	0.95 (0.57–1.60) *p* = 0.829	1.00^$^ (0.57–1.78) *p* = 0.996	82.6	0.74 (0.35–1.54) *p* = 0.419	1.15^$^ (0.53–2.52) *p* = 0.719	61.3	0.62^$^ (0.39–1.00) ***p* = 0.049**	0.82^$^ (0.49–1.36) *p* = 0.817
HPV-/p53Mut	49.3	1.0	1.0	77.7	1.0	1.0	45.6	1.0	1.0
HPV + /p53Wt	14.8	0.27 (0.10–0.76) ***p* = 0.013**	0.15^$^ (0.05–0.47) ***p* = 0.001**	91.8	0.33 (0.08–1.39) *p* = 0.130	0.42^$^ (0.08–2.23) *p* = 0.310	73.3	0.41^$^ (0.19–0.90) ***p* = 0.025**	0.72^$^ (0.29–1.78) *p* = 0.480
HPV + /p53Mut	56.2	0.56 (0.14–2.31) *p* = 0.422	0.65^$^ (0.15–2.81) *p* = 0.987	72.9	1.16 (0.28–4.92) *p* = 0.838	1.62^$^ (0.37–7.15) *p* = 0.526	42.2	1.41^$^ (0.61–3.27) *p* = 0.418	1.90^$^ (0.91–1.02) *p* = 0.184
	*p* = 0.056			*p* = 0.386			***p* = 0.021**		

*KM* Kaplan Meier analysis, *HR* Hazard Ratio, *p*-values below 0.05 are stated in bold

^*^Adjusted for age, FIGO status, growth pattern, invasion depth, and p53 status

^#^Adjusted for age, FIGO status, HPV status

^$^Adjusted for age, FIGO status, growth pattern, and invasion depth

The 5- and 10-year local recurrence rates and DSS did not differ in patients with p53 wildtype tumors compared to p53 mutated tumors (see Table 3). A better overall survival was found for patients with a p53 wildtype tumor compared to p53 mutation tumors (HR 1.85 (95% CI 1.21–2.84) *p* = 0.005. Adjusted for age, FIGO, and HPV status, this difference did not remain significant (OS (HR 1.47 (95% CI 0.92–2.34), *p* = 0.108)).

Subgroup analyses were performed based on HPV and p53 status: (1) HPV-p53Wt, (2) HPV-p53Mut, (3) HPV + p53Wt, and (4) HPV + p53Mut. Fewer local recurrences were found in patients in the HPV + /p53Wt (HR 0.15 (95% CI 0.05–0.47) *p* = 0.001) compared to the HPV-/p53Mut group, adjusted for age, FIGO status, growth pattern, and invasion depth (see Table 3). No differences were found for DSS survival within the HPV/p53 subgroups. Patients with HPV-/p53Wt (HR 0.62 (95% CI 0.39–1.00) *p* = 0.049) and with HPV + /p53Wt had better overall survival compared to patients with an HPV-/p53Mut status (see Table 3), which did not remain significant in multivariable analyses.

A slight difference was also found between p53Wt and p53Mut patients within the HPV-patient group, whereas HPV-/p53Mut had an inferior OS compared to HPV-/p53Wt (HPV-/p53Wt HR 1.0, HPV-/p53Mut 1.61 (95% CI 1.00–2.60) *p* = 0.049) (Supplementary Fig. 1). In multivariable analyses, adjusted for age, FIGO stage, growth pattern, and invasion depth, these results were not statistically significant (respectively HR 1.38 (95% CI 0.56–3.41) *p* = 0.480 and HR 1.22 (95% CI 0.73–2.04) *p* = 0.439).

Comparing p53 mutant and wildtype within the HPV tumors, there is a significant difference in the presence of perineural invasion (*p* = 0.001); for all other variables tested (LVSI, grade of differentiation, FIGO stage, p16 status, tumor diameter, depth of invasion, tumor-free margin, age at primary treatment, and time to first local recurrence), no differences were found.

## Discussion

In this study, we showed that local recurrence rates were higher in patients with HPV-negative compared to HPV-positive tumors; 10-year local recurrence rates were 48% versus 25% (*p* < 0.001). Local recurrence did not differ comparing p53 wildtype to p53-mutated tumors. DSS and OS did not differ between the analyzed groups based on HPV-PCR, p16 and p53 status, or a combination after multivariable analyses. It was also shown that p16 immunohistochemistry is a reliable surrogate marker for HPV status with a sensitivity of 86.4% and a specificity of 93.8%.

Patients with HPV-negative tumors have more local recurrences compared to patients with HPV-positive tumors and in univariable analyses also inferior OS. Currently, there is no difference in treatment and follow-up between patients with HPV-negative or positive VSCC. Given this difference in local recurrence and survival, determining HPV status (either by HPV-PCR or p16 IHC) could be key in future studies on the importance of follow-up, expectation management, and in future treatment regimens. As there are no clinical consequences for HPV status of the vulvar carcinoma, we advise not to perform an HPV-PCR or p16 IHC routinely. Within the context of clinical trials, we advise to perform an HPV-PCR or p16 IHC staining in all patients with VSCC.

In head and neck cancer, a distinction is made between HPV-positive and -negative carcinomas. Compared to HPV-negative cancers, HPV-positive cancers appear to be more responsive to chemotherapy and radiotherapy [28]. In vulvar cancer, one retrospective study, including patients receiving radiotherapy for VSCC, reported better progression-free survival, better overall survival, and lower in-field relapse rates in women with p16 or HPV-positive VSCC compared to p16 or HPV-negative VSCC [29]. These results suggest a more favorable radiation treatment response for HPV-positive VSCC and might contribute to future tumor-based treatment regimens. In our study, only patients with primary surgical treatment were included. In future studies, the response on radiotherapy in relation to HPV status can be assessed.

Follow-up for patients after treatment of VSCC is performed to detect locoregional recurrences as early as possible. Due to lack of prospective study data, an optimal surveillance strategy has not been established yet and follow-up regimens differ per guideline. Advised follow-up protocols vary from visits every 3 to 6 months during the first 2 years, every 6 to 12 months for the third, fourth, and fifth year, and all protocols recommend annual follow-up thereafter [30–33]. Since most groin recurrences present in the first 2 years after primary treatment, high frequent follow-up during those months is important [34]. Identification of high- and low-risk patient groups may lead to individualized follow-up schedules in the future. HPV status might be a parameter to distinguish these groups, because of the significant difference in 10-year local recurrence rate (24.6% for HPV-positive patients compared to 47.5% for HPV-negative patients). Further research is needed to establish a follow-up schedule based on low- and high-risk patient groups.

In this current study, we showed that p16 IHC has a sensitivity of 86.4% and sensitivity of 93.8% for HPV status. Therefore, p16 IHC is an accessible way to reflect the HPV status of a VSCC. These findings are in line with the results Rakislova et al. found in a large cohort of 1594 VSCC patients [6]. A good interpretation of the p16 IHC is of importance but can be challenging in some cases. For example, in cases with borderline or unclear p16 expression, in these cases, an HPV-PCR should be considered to give a more definitive answer about the HPV status of the tumor.

The performance of p53 IHC has no additional value in terms of prognosis. In our study, mutations of p53 were present in 49% of the patients of which the majority was present in HPV-negative tumors. Nooij et al. [14] identified three different subgroups with different prognosis (for local recurrence; HPV + , HPV-p53Wt, HPV-p53Mut). We could not reproduce these results in this study with a large series of VSCC patients.

We found in 4% of all patients an HPV + /p53Mut status. In addition, Nooij et al. [14] showed a TP53 mutation in only two (5%) HPV-positive VSCC. Suggesting that TP53 mutations, although rare, can also be seen in HPV-positive VSCCs.

Our study includes a large well-defined series of patients with VSCC with a long follow-up. Pathological review and assessment of immunohistochemical staining is performed in a structured way by two expert pathologists. Furthermore, our study is performed in accordance with the Reporting Recommendations for Tumor Marker Prognostic Studies (REMARK) rules [19].

Our study has two main limitations. Firstly, only unifocal VSCC was included; this might have introduced bias as HPV-related VSCC is more likely to be multifocal. Secondly, we used IHC to reflect the p53 mutation status, instead of TP53 mutation analyses. Currently, mutation analysis is prohibitively expensive and IHC is 95% correlated to mutation analysis [15, 16].

In conclusion, patients with HPV-positive tumors have a favorable prognosis in terms of fewer local recurrences compared to HPV-negative tumors. IHC-based analysis of p53 mutations had no additional value in predicting local recurrence. The HPV-negative tumors harbor the majority of p53 mutations. p16 status is a reliable surrogate marker for the detection of HPV presence. We advise to perform an HPV-PCR or p16 IHC staining in all patients with VSCC in daily clinical practice and in clinical trials to further develop tumor-based treatment and follow-up regimens.

### Supplementary Information

Below is the link to the electronic supplementary material.

Supplementary file1 (PNG 119 KB)
