# Supplementary figures and images for: Prognostic value of HPV-PCR, p16 and p53 immunohistochemical status on local recurrence rate and survival in patients with vulvar squamous cell carcinoma
Source: Virchows Arch. 2023 Nov 8;484(6):985–94. doi: 10.1007/s00428-023-03690-8 (PMC11186908; doi:10.1007/s00428-023-03690-8)

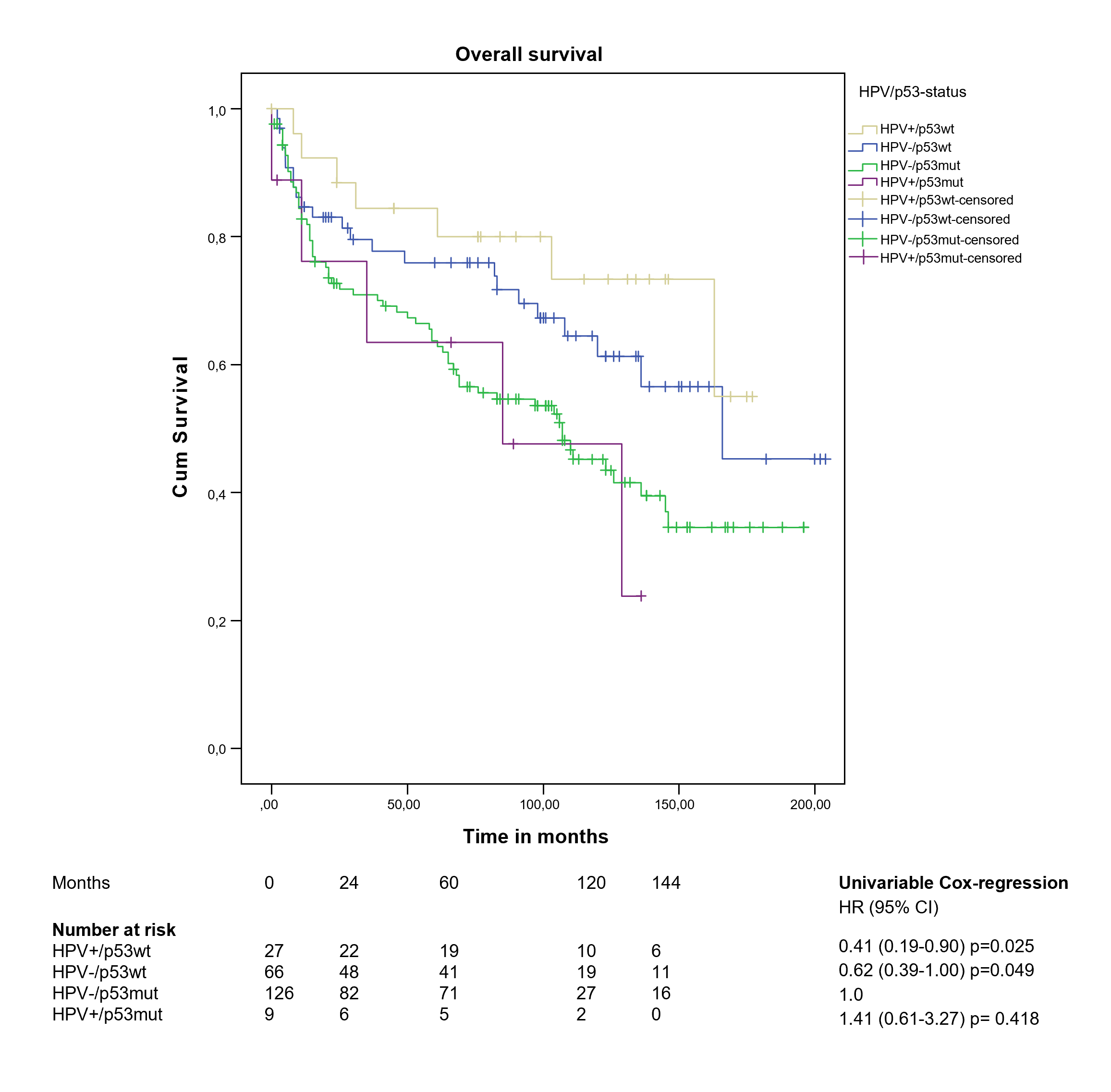

Supplement: Supplementary file 1 — Supplementary file1 (PNG 119 KB) [file 428_2023_3690_MOESM1_ESM.png]
